# CT引导下穿刺活检对Ⅰ期-Ⅱ期非小细胞肺癌远处转移和生存的影响

**DOI:** 10.3779/j.issn.1009-3419.2017.03.07

**Published:** 2017-03-20

**Authors:** 景丽 范, 可可 翟, 婷婷 任, 晓 冯, 林 隋, 晶 胡, 庆威 孟

**Affiliations:** 1 150000 哈尔滨，哈尔滨医科大学附属肿瘤医院肿瘤内科 Department of Medical Oncology, Harbin Medical University Cancer Hospital, Harbin 150000, China; 2 150000 哈尔滨，哈尔滨医科大学附属肿瘤医院影像科（隋林） Department of Medical Imaging, Harbin Medical University Cancer Hospital, Harbin 150000, China

**Keywords:** 肺肿瘤, CT引导下穿刺活检, 活检, 远处转移, 生存, Lung neoplasms, Computed tomography-guided percutaneous needle biopsy, Biopsy, Distant metastasis, Survival

## Abstract

**背景与目的:**

CT引导下穿刺活检（computed tomography-guided needle biopsy, CTNB）是诊断肺癌最常用的一种方法，具有诊断准确性高及并发症少的特点。现有的研究表明CTNB可引起肿瘤的针道转移，然而极少有研究关注CTNB对远处转移和远期生存的影响。本研究旨在探讨术前的CTNB对Ⅰ期-Ⅱ期非小细胞肺癌（non-small cell lung cancer, NSCLC）远处转移和生存的影响。

**方法:**

研究对象为1, 234例Ⅰ期-Ⅱ期NSCLC术后患者，其中113例术前行CTNB，1, 121例术前未经任何活检。应用倾向性评分匹配方法平衡两组间的临床特征，经配对后纳入后续统计检验共226例。并进一步应用*Cox*回归分析法和*Kaplan*-*Meier*分析法进行生存分析。

**结果:**

在Ⅰ期-Ⅱ期NSCLC患者中，术前的CTNB组无远处转移时间（distant metastasis free survival, DMFS）显著短于无活检组（*P*=0.032），而两组间总生存无统计学差异（*P*=0.086）。

**结论:**

CT引导下穿刺肺活检可能促进早期肺癌的远处转移，但不影响总生存。

## Abstract (en)

**Background and objective:**

Computed tomography-guided needle biopsy (CTNB) is a well-established and commonly used technique for diagnosing pulmonary nodules with high accuracy and safety. Needle-tract implantation after CTNB has been reported in various reports. The aim of this study is to evaluate whether preoperative CTNB affected the distant metastasis and overall survival in pathological stage Ⅰ-Ⅱ lung cancer patients.

**Methods:**

A total of 1, 234 patients with pathological stage Ⅰ-Ⅱ non-small cell lung cancer were collected. 113 patients received preoperative CTNB, and 1, 121 patients did not receive any biopsy before surgical resection. Propensity score-matching method was adopted to balance observed covariates between two groups, 113 pairs were matched. *Cox* regression analysis and *Kaplan*-*Meier* estimates were used to process survival analysis.

**Results:**

Distant metastasis free survival (DMFS) was significantly poorer in the preoperative CTNB group than in the non-biopsy group (*P*=0.032). But there was no difference in the overall survival between the two groups (*P*=0.086).

**Conclusion:**

CTNB increased the risk of distant metastasis, not increasing the risk of mortality.

非小细胞肺癌（non-small cell lung cancer, NSCLC）是肺癌最常见的组织学类型，约占肺癌总数的80%-85%。近年来其发病率不断增高，严重危害人类身心健康，早期诊断至关重要。目前常用的诊断方法为CT引导下穿刺活检（computed tomography-guided needle biopsy, CTNB）。CTNB具有诊断准确性高及并发症少的特点^[1-5]^。其最常见的并发症是气胸和咯血^[6-8]^，但一般是轻度的，且易于处理。另外一个少见的并发症是恶性肿瘤的针道转移^[9-12]^，然而由于穿刺针的改进，已经进一步大大减少了针道转移的发生率。临床上仍令操作者和患者担心的是，穿刺是否会增加血行转移的机会、是否会影响生存？这在之前的研究中很少被提到。因此，我们回顾性研究了术前CT引导下穿刺活检对Ⅰ期-Ⅱ期NSCLC远处转移和生存的影响。

## 1 材料与方法

### 1.1 临床资料

选取哈尔滨医科大学附属肿瘤医院2011年1月-2014年12月间行开胸根治性手术切除的Ⅰ期-Ⅱ期NSCLC患者，术后根据第7版肿瘤-淋巴结-转移（tumor-node-metastasis, TNM）分期系统进行病理分期。排除伴有其他恶性肿瘤、术前行支气管镜活检、非根治性手术或术后即失访的患者。最终，1, 234例患者符合要求。生存时间从术后第1天算起，截止至2016年08月13日。中位随访31.15个月（0.17个月-68.70个月）。

### 1.2 倾向性评分匹配

倾向性评分匹配是一种从原始数据中挑选出与实验组相似的对照组数据的方法，其适用于回顾性研究，以期最大程度消除不同组间协变量的不均衡^[13]^。除前瞻性随机对照试验之外，在回顾性对照研究中它可以提供最匹配的对照组合。在这1, 234例患者中，113例术前经过CT引导下穿刺活检确诊作为穿刺组（观察组），1, 121例经术中确诊作为无穿刺组（对照组）。采用1:1的匹配模型进行倾向性评分匹配，观察组113例均得到匹配，匹配后两组间的临床特征无统计学差异（表 1）。倾向性评分匹配采用R语言2.15软件包实现。

**1:** 患者的临床资料 Clinical characteristics of patients

Clinical factors	Overall cohort (*n*=1, 234)				Propensity score-matched cohort (*n*=226)		
	CTNB (*n*=113)	Non-biopsy (*n*=1, 121)	*P*		CTNB (*n*=113)	Non-biopsy (*n*=113)	*P*
Age (yr)			0.698				0.690
≥60	57	544			57	60	
＜60	56	577			56	53	
Gender			0.791				0.894
Male	58	590			58	59	
Female	55	531			55	54	
T stage			＜0.001				0.396
1	30	586			30	27	
2	78	502			78	76	
3	5	33	3			10	
N stage			0.448				0.857
0	94	962			94	95	
1	19	159			19	18	
TNM stage			＜0.001				0.087
Ⅰ	71	890			71	83	
Ⅱ	42	231			42	30	
Location			0.319				0.391
Central	8	112			8	5	
Peripheral	105	1, 009			105	108	
Pathology			0.001				0.840
Ad	78	919			78	82	
Scc	24	162			24	21	
Others	11	40			11	10	
Chemotherapy^#^			＜0.001				0.506
Yes	57	275			57	52	
No	56	846			56	61	
Radiotherady^#^			0.143				0.313
Yes	3	12			3	1	
No	110	1, 109			110	112	
TNM: tumor-node-metastasis; Ad: adenocarcinoma; Scc: squamous-cell carcinoma; CTNB: computed tomography-guided needle biopsy. #: postoperative adjuvant therapy.							

### 1.3 穿刺技术

所有CT引导下穿刺肺活检均由具有10年以上工作经验的影像科医师操作，严格遵守无菌及操作流程执行。所用的穿刺针均为HS医院服务股份公司生产的型号PRECISA 18 GX150MM的穿刺针。

### 1.4 研究终点

本研究主要的研究终点包括无远处转移生存时间（distant metastasis free survival, DMFS）和总生存时间（overall survival, OS）。远处转移定义为出现以下情况中任一项：①恶性胸腔或心包积液；②胸膜转移；③对侧肺转移；④出现胸腔外的转移。DMFS定义为从手术到第一次出现远处转移或能够确定未发生远处转移的末次随访的时间。OS定义为从手术到任何原因死亡或末次随访的时间。

### 1.5 统计学处理

采用SPSS 22.0进行统计学分析。率的比较采用*χ*^2^检验，生存分析采用*Kaplan*-*Meier*生存曲线和对数秩检验，单因素和多因素分析采用*Cox*回归分析模型，以*P*＜0.05为差异有统计学意义。

## 2 结果

### 2.1 患者临床特点分析

本研究中共有1, 234例患者，经倾向性评分匹配后共226例患者匹配成功，其临床特征见表 1。其中，年龄分为≥60岁和＜60岁两组，只有病理为分类变量，余为连续变量。经匹配后两组间的临床特征没有统计学差异。匹配后数据中，至末次随访时，CTNB组中远处转移39例（34.51%），死亡29例（25.66%）。而无活检组中远处转移25例（22.12%），死亡19例（16.81%）。最常见的转移部位为脑，其次为骨、肺内、肝脏等转移部位。

### 2.2 生存分析

匹配前数据经*Kaplan*-*Meier*生存曲线分析显示，与无穿刺组相比，穿刺组的DMFS（*P*＜0.001）和OS（*P*=0.002）均明显降低。匹配后数据经*Kaplan*-*Meier*生存曲线分析显示，与对照组相比，观察组的DMFS较短，差异具有统计学意义（*P*=0.032）。两组间的OS差异无统计学意义（*P*=0.086）（图 1）。

**1:**
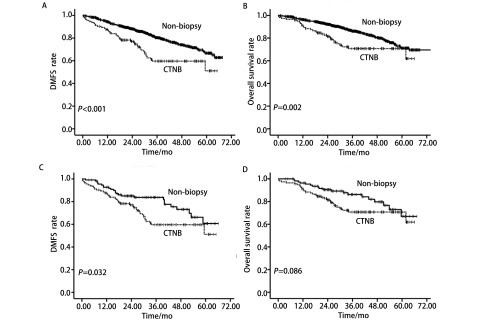
各组DMFS和OS情况。A：在整体数据中，与对照组相比，观察组的DMFS明显缩短（*P*＜0.001）；B：在整体数据中，与对照组相比，观察组的OS明显缩短（*P*=0.002）；C：在匹配后数据中，与对照组相比，观察组的DMFS明显缩短（*P*=0.032）；D：在匹配后数据中，两组间的OS无明显差异（*P*=0.086）。 DMFS and OS rates in patients with stage Ⅰ-Ⅱ lung cancer. A: DMFS was significantly reduced in the CTNB group as compared to the non-CTNB group (CTNB group, *n*=113; non-CTNB group, *n*=1, 121; *P* < 0.001, *Log*-*rank* test); B: OS was significantly reduced in the CTNB group as compared to the non-CTNB group (CTNB group, *n*=113; non-CTNB group, *n*=1, 121; *P*=0.002, *Log*-*rank* test); C: DMFS was significantly reduced in the CTNB group as compared to the non-CTNB group in the matched cohort (CTNB group, *n*=113; non-CTNB group, *n*=113; *P*=0.032, *Log*-*rank* test); D: OS was similar for the two groups in the matched cohort (CTNB group, *n*=113; non-CTNB group, *n*=113; *P*=0.086, *Log*-*rank* test). DMFS: distant metastasis free survival; OS: overall survival.

### 2.3 *Cox*回归分析

*Cox*单因素分析发现N分期、病理类型、穿刺活检、放疗均与患者的DMFS相关。将T分期、N分期、病理类型、穿刺情况、辅助放疗、辅助化疗均纳入*Cox*多因素分析模型，采用逐步向后法，结果显示只有病理类型、N分期和穿刺活检是肺癌患者DMFS的独立危险因素。同样地，关于患者的临床特征与总生存的关系也做了*Cox*回归分析。*Cox*单因素分析发现N分期、病理类型、放疗均与患者的OS相关，将T分期、N分期、病理、穿刺活检、辅助放疗、辅助化疗均纳入*Cox*多因素分析模型，结果显示只有N分期和病理是患者的独立预后危险因素（表 2）。

**2:** 单因分析和多因素分析预测DMFS和OS的影响因素 Univariable and multivariable analyses of predictors of DMFS and OS for propensity score-matched cohort

Clinical factors	Univariable analysis of DMFS				Univariable analysis of OS		
	HR	95%CI	*P*		HR	95%CI	*P*
Age (≥60 yr *vs*＜60 yr)	1.057	0.646-1.730	0.824		1.515	0.844-2.718	0.164
Gender (female *vs* male)	0.815	0.497-1.337	0.418		0.630	0.351-1.132	0.122
T stage	1.202	0.770-1.877	0.419		1	0.592-1.690	1.000
N stage	2.387	1.380-4.127	0.002^*^		2.236	1.198-4.175	0.011^*^
Location (peripheral *vs* central)	0.645	0.259-1.608	0.347		0.640	0.230-1.781	0.640
Pathology							
Scc *vs* Ad	0.363	0.191-0.690	0.002^*^		0.386	0.184-0.812	0.012^*^
Others *vs* Ad	0.326	0.141-0.758	0.009^*^		0.390	0.150-1.013	0.053^*^
Chemotherapy^#^	1.445	0.876-2.384	0.250		0.822	0.466-1.452	0.500
CTNB or non-biopsy	1.720	1.041-2.844	0.034^*^		1.652	0.951-2.948	0.090
Radiotherapy^#^	3.829	1.194-12.280	0.024^*^		3.891	1.205-12.568	0.023^*^
^*^P＜0.05; ^#^: postoperative adjuvant therapy.							

## 3 讨论

CT引导下穿刺肺活检是诊断肺癌最常用的一种方法。曾有研究证实其可引起针道的转移，虽然发生率极低。本研究对113例术前行CT引导下穿刺肺活检及1, 121例术前未行任何穿刺活检的Ⅰ期-Ⅱ期NSCLC患者进行回顾性分析，发现CT引导下穿刺肺活检不增加患者的死亡风险（*P*=0.086）。尽管没有达到统计学意义，但是我们的数据仍然显示穿刺组患者有较高死亡风险的趋势。强烈提示我们存在如下可能性：当样本量扩大时两组之间的差异会具有显著性。Wisnivesky等^[14]^曾回顾性分析8, 607例Ⅰ期NSCLC患者，认为经皮穿刺肺活检不影响Ⅰ期NSCLC患者的总生存（*P*=0.570）。与之前的研究相比，本研究中的对照组是没有经过任何术前活检的，而在前者研究中两组中均含有大量支气管镜活检的患者。我们不确定支气管镜活检是否也会增加患者的死亡风险。

重要的是，我们还观察到CT引导下穿刺肺活检增加患者的远处转移风险（*P*=0.032）。类似的，Kashiwabara等^[15]^在149例Ⅰ期NSCLC患者中观察到，术前经皮肺穿刺会增加胸膜转移的机会。虽然该作者仅仅观察胸膜转移的情况，但仍然提示经皮肺穿刺增加血行转移的机会。而Asakura等^[16]^在321例Ⅰ期NSCLC患者中观察到，术前的经皮肺穿刺并没有增加胸膜转移的机会。但此研究中，相比于对照组，穿刺组含有更多的P-T1a患者。我们的研究强烈提示对早期肺癌患者的经皮肺穿刺会增加远处转移的机会。因此，对临床早期肺癌患者来说，穿刺活检应该慎重选择。

与以往研究相比，我们的研究中不包含经支气管镜肺活检等有创检查。另外，我们在统计分析过程中采用倾向性评分匹配方法，最大程度减少了混杂因素的影响。因此，我们的研究在先前的研究基础之上，补充了新的数据。虽然我们的研究不足以改变现有的临床实践模式，但提示关于穿刺活检在恶性肿瘤中的应用值得更多的关注。
